# Tuberculosis and Cardiovascular Complications: An Overview

**DOI:** 10.7759/cureus.28268

**Published:** 2022-08-22

**Authors:** Mayowa A Adefuye, Nisha Manjunatha, Vinutna Ganduri, Kruthiga Rajasekaran, Shrimahitha Duraiyarasan, Bolanle O Adefuye

**Affiliations:** 1 Research, University of Ibadan College of Medicine, Ibadan, NGA; 2 Research, Our Lady of Fatima University College of Medicine, Valenzuela, PHL; 3 Research, Bhaskar Medical College, Hyderabad, IND; 4 Research, Rajah Muthiah Medical College & Hospital, Chidambaram, IND; 5 Research, K.A.P. Viswanatham Government Medical College, Tiruchirappalli , IND; 6 Pulmonary Medicine, Obafemi Awolowo College of Health Sciences, Olabisi Onabanjo University, Sagamu, NGA

**Keywords:** tuberculous aortitis, tuberculous myocarditis, tuberculous pericarditis, cardiovascular complications, tb, tuberculosis

## Abstract

Tuberculosis (TB) is a dominant cause of mortality from a single infectious disease agent. It is a global health issue that has been tagged as a public health emergency for decades. The disease process, which is caused by *Mycobacterium tuberculosis* (MTB), affects the respiratory system as well as many other organ systems in the body, such as the lymphatic system, central nervous system (CNS), gastrointestinal system, and cardiovascular system (CVS). Generally, cardiovascular diseases are the leading cause of death worldwide, with most of the mortality in low and middle-income countries. Also, the high mortality rate of TB is skewed to these regions, making the mortality of TB with CVS involvement exceptionally high.

The multisystemic involvement of TB impacts the cardiovascular system in various forms. While pericarditis caused by TB is quite common, other complications like myocarditis, coronary artery disease, and aortitis are rarer, necessitating a high index of suspicion and holistic management.

This article reviews the pathophysiology of cardiovascular complications in TB, highlighting mechanisms of occurrence, common complications, management protocols, and prognostic factors. Our review highlights some of the gaps in understanding cardiovascular complications in TB, necessitating further research to investigate causal mechanisms and treatment.

## Introduction and background

Tuberculosis (TB) remains a global health problem, with its discovery dating back centuries [1]. It has been postulated that the causal genus, *Mycobacterium*, emerged over 150 million years ago [2]. Thus, over the ages, different names have been ascribed to the disease, from *"*schachepheth” by the ancient Hebrews to “phthisis”, *“*consumption”, and “king’s evil” by the ancient Greeks, the English, and the French, respectively [3,4]. Globally, it is one of the leading causes of mortality from a single infectious disease agent, with the mortality rate increasing exponentially in those with severe immunosuppressive disorders [5]. According to the World Health Organization (WHO) Global Tuberculosis Report 2021, 9.9 million people were infected with TB in 2020, with 1.3 million people dying, which is 13% of the total number infected [6]. Of these, a higher prevalence was seen in South-East Asia, Africa, and the Western Pacific regions, with a global male-to-female ratio of 1.7:1 [6]. Although there has been a significant reduction in TB-related mortality over the last three decades due to improvements in antimicrobials and public health measures, current figures are still alarming, and better control measures need to be implemented, especially in light of the coronavirus disease 2019 (COVID-19) pandemic.

TB is caused by the microorganism *Mycobacterium tuberculosis* (MTB), an airborne infectious agent that can rapidly spread from person to person during its active phase [7]. Immunosuppressive diseases such as diabetes mellitus (DM) and human immunodeficiency virus (HIV), as well as excessive alcohol intake, tobacco smoke, and indoor air pollution, have been shown to accelerate disease progression [8]. A study by Shimeles et al. in Ethiopia identified illiteracy, low household income, and lack of a Bacille Calmette-Guérin (BCG) scar as independent risk factors [9]. This echoes the socioeconomic problems experienced in many developing countries as it contributes to overcrowding and malnutrition, among other things. The lung is usually the primary site of infection, and common presenting symptoms include chronic cough, fever, hemoptysis, weight loss, night sweats, and loss of appetite [7]. As the disease progresses, it can spread to involve multiple organ systems, such as the lymphatic system, central nervous system (CNS), gastrointestinal (peritoneum commonly), and cardiovascular system (CVS), amongst others [10]. The lymph nodes are the most frequently involved, and the presence of necrotic lymph nodes increases the diagnostic likelihood of extrapulmonary involvement [10]. Ohene et al. found that of 3,342 patients diagnosed with TB, 21.8% had extrapulmonary tuberculosis (EPTB) [11]. This percentage is daunting and mirrors findings in similar studies; thus, a closer look has to be taken at EPTB.

As far back as the early 1900s, Pottenger had noted the effects of TB on the CVS; even now, in the modern era, mortality rates of those with CVS involvement approach 60% [11,12]. Globally, cardiovascular diseases (CVDs) are the leading cause of death; thus, intersections of TB and CVS, especially with such high mortality rates, should be closely examined [13]. Cardiovascular complications of TB are one of the commonest extrapulmonary involvements of the disease. These complications from TB are numerous, with pericarditis showing a high prevalence. Others are acute myocardial infarction (AMI), aortitis, myocarditis, and mycotic aneurysms [14,15].

This review provides a comprehensive overview and discussion of the cardiovascular complications of tuberculosis, its pathophysiology, and management.

## Review

Pathophysiology of cardiovascular involvement in TB

Traditionally, the most common risk factors associated with the development of CVDs are conditions such as hypertension, DM, obesity, hyperlipidemia, and smoking; however, studies have linked infection as contributing to the development of CVDs [16,17]. Several case studies and population-based studies that show a direct relationship between TB and cardiovascular complications have been published, and different theories have been postulated regarding the mechanisms of occurrence [10,11].

The monocytes, macrophages, cytokines, and lymphocytes, which propagate cell-mediated responses against MTB are vital factors that drive atherogenesis [18]. Thus, as the body tries to protect itself by fighting the causative agent, it harms itself in the same process. The persistent systemic inflammation leads to atherosclerotic plaque formation in vessels [18]. Some postulation has been made on MTB inhabiting the atherosclerotic plaques and directly damaging vasculature [16].

Molecular mimicry and autoimmunity are other critical components hypothesized in the pathophysiology of cardiovascular complications in TB. This involves the heat shock protein (HSP) system [19]. About 40-50% of residues in human HSP65 and MTBHSP65 are identical. Thus, exposure to infection or other stressors induces expression of HSP65 on the surface of endothelial cells, and this results in a cross-reaction between antibodies produced against MTB HSP65 and self-antigens HSP60 in the host [19,20]. Xu et al. conducted a study on 120 white rabbits with normal serum cholesterol; they were grouped and inoculated with six antigens and adjuvants, one of which was HSP65. The rabbits were then killed 16 weeks after primary inoculation, and atherosclerotic plaques were found only in the sub-set inoculated with whole mycobacteria/recombinant HSP65 even though their serum cholesterol levels remained normal [21]. A later study done by Zhu et al. on 201 asymptomatic human subjects found an association between HSP65 antibodies and elevated levels of coronary calcification [22]

Though these have been elaborated on, the precise mechanisms of certain cardiovascular complications in TB remain unknown.

Tuberculous pericarditis

Tuberculous pericarditis is a major cause of pericardial disease globally, with its incidence higher in developing regions of the world. It occurs in approximately 1-2% of cases of TB [23,24]. Reuter et al. conducted a prospective study in South Africa that spanned six years; of the 233 patients with pericardial effusion, MTB was the causative agent in 69.5% (Table 1) [25]. A comparable study, also in South Africa, was published almost a decade after. It was a retrospective study by Mutyaba et al. in which medical records of patients who had undergone pericardiectomy on account of constrictive pericarditis were reviewed. There were a total of 121 patients over the 22 years reviewed, and 90.9% were attributed to TB. TB was proven in 29.8%, and presumed in 61.2%; proven TB was classified as a case in which microscopy or culture of pericardial tissue, fluid, or sputum isolated MTB, while those with a positive history of TB or who had been commenced on anti-TB medications before pericardiectomy was defined as a case of presumed TB (Table 1) [26].

**Table 1:** Principal causes of pericarditis in populations across various regions

Reference	Design	Sample Number	Population	Conclusion
Reuter et al., 2005 [25]	Prospective study	233	Patients between the ages of 13-85 years found to have large pericardial effusion, South Africa	Tuberculosis was the commonest cause of pericarditis in South Africa.
Mutyaba et al., 2014 [26]	Retrospective review of medical records	121	Patients between the ages of 14-74 years who underwent pericardiectomy on account of constrictive pericarditis, South Africa	Tuberculosis was the most predominant cause of constrictive pericarditis in South Africa.
Pio et al., 2016 [27]	Prospective and longitudinal study	38	Patients between the ages of 16-73 years with effusive pericarditis, Lomé Togo	Tuberculosis was the leading cause of effusive pericarditis.
Karima et al., 2021 [28]	Longitudinal and retrospective bicentric study	25	Patients between the ages of 7-72 years diagnosed with constrictive pericarditis, Tunisia	Post-tuberculosis constrictive pericarditis was predominant and associated with right ventricular dysfunction. Treatment with pericardiectomy was effective.
Gouriet et al., 2015 [29]	Prospective cohort study	1162	Patients between the ages of 3 months-93 years diagnosed with acute pericarditis	The leading cause of pericarditis was post-injury syndrome. There was a reduction in the percentage of idiopathic cases as the etiology was found for most cases.

Another study was conducted in Lomé, West Africa, by Pio et al. in 2016. It was a prospective longitudinal study on 38 patients hospitalized with effusive pericarditis. On the evaluation of the causative agent, TB was identified as the leading cause of effusive pericarditis accounting for 55% of cases (Table 1) [27]. Similar results were found in Tunisia, where TB was the commonest cause of constrictive pericarditis, in 44% of cases (Table 1) [28].

The above statistics differ from the study by Gouriet et al. in France. It was a prospective cohort study on the etiology of pericarditis in 1162 cases. In the study, only 5.7% of cases were caused by an infection, and this was mainly attributed to staphylococcal infection and Lyme disease; there were only a few cases of TB diagnosed, and a precise percentage was not stated (Table 1) [29]. This result mirrors findings in other developed climes of the world, where TB is only responsible for about 4% of cases of pericarditis [30].

Patients with tuberculous pericarditis can present in various forms: a pericardial effusion, constrictive pericarditis, or a constriction-effusion presentation [31]. Clinical symptoms include fever, malaise, chest pain, cough, breathlessness, night sweats, weight loss, and right upper quadrant pain due to liver congestion. Some of these symptoms listed show that their presentation can be similar to that of heart failure (as it can lead to this). As such, proper assessment of each case is essential to find the precise etiology to ensure appropriate and effective management of patients. Signs on physical examination may include tachycardia, hypotension, pulsus paradoxus, pericardial knock, pericardial friction rub, distant or muffled heart sounds, hepatomegaly, ascites, and edema [32].

Diagnosis of pericardial effusion or constrictive pericarditis can be made via echocardiography, CT, and MRI [31]. Though these can be used to visualize the fluid collection, pericardial thickening, or calcification, invasive procedures such as pericardiocentesis are often needed to determine the cause. The presence of cardiomegaly on a chest radiograph can be suggestive of pericardial effusion; and in patients with tuberculous pericarditis, the chest radiograph may also show features suggestive of TB, such as nodules, cavitation, consolidation, and hilar lymphadenopathy. When tuberculous pericarditis is suspected, sputum collection and pericardiocentesis are recommended to enable isolation of the acid-fast bacilli via microscopy and culture or Xpert MTB/RIF assay [24,31]. Elevated pericardial adenosine deaminase (ADA) activity and increased interferon-γ (IFN- γ) levels in pericardial fluid are also highly suggestive of tuberculous pericarditis, and are less time-consuming when compared to culture, but more costly. A pericardial biopsy and subsequent histology can also be used for diagnosis [31].

The treatment goals of tuberculous pericarditis are to treat active TB, relieve symptoms of constriction or effusive process and prevent progression to complications. Before the introduction of anti-TB medications, mortality from tuberculous pericarditis was about 80-90%; this has significantly reduced now to about 26% in some populations [33,34]. Treatment involves the commencement of anti-TB medications comprising rifampicin, isoniazid, pyrazinamide, and ethambutol [35]. These medications have been found to poorly penetrate the pericardium, which explains the high mortality rates still seen [35]. More research should be done to investigate alternative medications and possible dose alterations. Pericardiocentesis, which was discussed above, also has a therapeutic function and alleviates symptoms in patients presenting with cardiac tamponade. A pericardiectomy may be indicated in patients with constrictive symptoms [31]. The use of corticosteroids in tuberculous pericarditis is controversial as studies have varying conclusions on its therapeutic efficacy. However, despite this, it is widely used [36-39]. Table 2 contains a summary of some studies conducted on the use of corticosteroids in tuberculous pericarditis.

**Table 2:** Use of corticosteroids in tuberculous pericarditis

Reference	Design	Sample Number	Population	Conclusion
Wiysonge et al., 2017 [36]	Systematic Review	1959	Data from seven trials conducted in Sub-Saharan Africa	Corticosteroid use in tuberculous pericarditis may reduce death in HIV-negative persons, and may reduce constriction in HIV-positive patients not on antiretroviral medications.
Mayosi et al., 2014 [37]	Randomized Control Trial	1400	Adults with definite or probable tuberculous pericarditis	There was no notable effect of prednisolone or mycobacterium indicus pranii on combined death, constrictive pericarditis, or cardiac tamponade requiring pericardiocentesis.
Reuter et al., 2006 [38]	Randomized Control Trial	57	Patients with massive tuberculous pericardial effusion requiring pericardiocentesis	Both systemic and intrapericardial corticosteroids were well tolerated but had no significant effect on clinical outcomes.
Strang et al., 2004 [39]	Randomized Double-blind Control Trial	383	Patients with tuberculous constrictive pericarditis and tuberculous pericardial effusion	A corticosteroid should be added to antituberculous medications when treating tuberculous pericarditis once contraindications are excluded.

Tuberculous myocarditis and sudden cardiac death

TB is a rare cause of myocarditis accounting for about 0.14-2% in some studies [40,41]. Many case reports show that the most common populace affected are young adults <45 years. There is also a male predominance with a male-to-female ratio of 2:1 [42]. Tuberculous myocarditis can occur together with pericarditis as myopericarditis or as an isolated disorder. Most cases of tuberculous myocarditis are asymptomatic, but some may present with conduction abnormalities such as ventricular arrhythmias and atrioventricular block. They may also present with dilated cardiomyopathy, congestive heart failure, or sudden cardiac death (SCD) [42,43]. Tuberculous myocarditis can occur from direct spread from the pericardium as implied above, retrograde spread from lymph nodes, or via hematogenous seeding [44]. Early diagnosis poses a challenge. This is likely due to its low incidence, asymptomatology, and higher prevalence in young adults. Despite these, awareness should be increased, especially in TB endemic regions. The mortality rate associated with symptomatic tuberculous myocarditis is high, with most cases diagnosed post-mortem [42].

When suspected in a patient, a transthoracic echocardiogram or cardiac MRI should be done. This would allow visualization of valvular or other mechanical abnormalities. ECG can help detect conduction abnormalities in symptomatic patients. Endomyocardial biopsy has been suggested to diagnose myocarditis of unclear etiology, but it weakly recommended by some guidelines [45,46].

There is limited data on standard treatment of tuberculous myocarditis beyond the commencement of anti-TB medications. This is likely due to the low incidence of the disorder. Though anti-TB medications may improve the immediate clinical condition, they do not reduce the lifetime risk of SCD; so patients should be monitored over time [42]. Management from case reports published is tailored to the individual presentation of the patient and complications that arise. Table 3 gives a summary of some case reports that have been published on tuberculous myocarditis [43,47-50].

**Table 3:** Summary of some case reports on tuberculous myocarditis SCD: sudden cardiac death; M: male; F: female

Reference	Age	Sex	Immunological Status	Pulmonary Involvement	Area of Heart Affected	Other Extrapulmonary Involvement	Outcome of Therapy
Cowley et al., 2017 [43]	33	M	Competent	Yes	Biventricular failure	Paratracheal, hilar, and mediastinal lymph nodes	Fatal (SCD)
Vennamaneni et al., 2022 [47]	29	M	Competent	Yes	Left ventricle and mitral valve		Fatal (SCD)
Choudhary et al., 2021 [48]	34	F	Competent	Yes	Left ventricle, mitral valve, and tricuspid valve	Lymphadenitis	Responsive
Amonkar et al., 2009 [49]	64	F	Not reported	-	Biventricular affectation with pericardial involvement	Liver	Fatal (SCD)
Kumar et al., 2019 [50]	5	F	Not reported	Not reported	Left ventricle	Cervical lymph nodes	Responsive

Tuberculous aortitis

Tuberculous aortitis is a rare cardiovascular complication of TB first described by Weigbert in 1882 [51]. It occurs in about <1% of cases and is usually suggestive of a disseminated spread [52]. About 50% are associated with aneurysms, and the descending thoracic and abdominal aorta are the most commonly affected. Although more patients present with aneurysms, some develop inflammatory aortic stenosis [53,54]. Patients commonly present with systemic TB symptoms and also with symptoms from aneurysmal mass effect, such as dysphagia and hoarseness, or sometimes hemodynamic instability due to rupture [54]. CT and CT angiography are recommended radiographic imaging modalities that allow a proper assessment of tuberculous aortitis. There should be a high index of suspicion in patients presenting with systemic TB symptoms and an aneurysm, especially in TB endemic regions [55]. Management usually involves both surgical resection of the aneurysm and aortic bypass grafting as well as a long course of anti-TB medications, sometimes for as long as a year. Though the overall prognosis is poor, with mortality approaching 50% in some studies, the prognosis is poorer when either medical therapy or aneurysm resection is attempted in isolation [51,56]. Some authors propose longer or life-long use of anti-TB medications to prevent complications such as prosthetic infections or anastomotic aneurysms [54,57]. Patients with inflammatory aortic stenosis are initially commenced on anti-TB medications with corticosteroids as an adjunct. Surgery is then considered if the clinical condition fails to improve [53].

Tuberculosis and coronary heart disease

The pathogenesis of TB-related atherosclerosis discussed in the pathophysiology sub-section of this review illuminates how TB and coronary heart disease (CHD) are interconnected. Furthermore, TB and CHD have been said to have epidemiologic similarities. From a pathological perspective, systemic inflammation plays a significant role in the development of CHD in TB, and this inflammation propels immunological activation, which drives the development of CHD. Also, the co-infection of HIV with TB, commonly seen in low and middle-income countries, further contributes to this immunological response leading to worse outcomes in patients [58,59].

A meta-analysis that evaluated four cohort studies with 83,500 TB cases was published in 2020. This study found the pooled relative risk of developing CHD in patients with TB to be 1.76 (95%CI = 1.05-2.95) compared to patients without TB (Table 4) [60]. Huaman et al. conducted another study investigating the relationship between latent TB and AMI. It was a case-control study in which 105 patients with AMI and 110 non-AMI controls were enrolled. The results showed a higher frequency of latent tuberculosis in AMI patients compared to non-AMI controls (Table 4) [59]. Other studies done by Hasanain et al. and Chung et al. found similar results with TB increasing the risk for CHD in both studies (Table 4) [61,62].

**Table 4:** Tuberculosis and coronary heart disease TB: tuberculosis

Reference	Design	Sample Number	Population	Conclusion
Wongtrakul et al., 2020 [60]	Systematic Review and Meta-analysis	83,500	Patients with TB and patients without TB	There was an increased risk of coronary heart disease in patients with TB.
Huaman et al., 2018 [59]	Case-Control	215	Patients with acute myocardial infarction and controls without acute myocardial infarction, Peru	Latent tuberculosis was an independent risk factor for acute myocardial infarction.
Hasanain et al., 2018 [61]	Case-Control	183	Patients with coronary artery stenosis and a control group without coronary artery stenosis, Egypt	Latent tuberculosis was a risk factor for coronary artery stenosis.
Chung et al., 2014 [62]	Population-based cohort study	50,840	Patients with TB and patients without TB, Taiwan	Patients with TB were at increased risk for acute coronary syndrome, with the risk increasing with advanced age.

A difference in the management of CHD in patients with TB and those without TB has not been discussed in the literature. Close attention should be paid to medications administered to prevent adverse drug-drug interactions with anti-TB medications when needed.

Tuberculosis and ischemic stroke

Ischemic stroke is a well-documented complication of tuberculous meningitis which has been broadly discussed. A retrospective review of 104 patients with tuberculous meningitis in New Zealand showed that stroke accounted for 33% of complications [63]. This is a similar picture seen in several studies that have noted an increased risk of stroke in patients diagnosed with tuberculous meningitis [64,65]. One can reason that direct spread and inflammation of vessels contribute to this, but it leaves us wondering if there is also an increased risk of stroke in patients who have TB without direct CNS involvement.

Some researchers have conducted studies to determine if there is an association between ischemic stroke and TB without CNS involvement. Sheu et al. conducted a retrospective cohort study published in 2009 where they investigated the risk of ischemic stroke over three years following the diagnosis of TB. The study included 2283 patients treated for TB and 6849 randomly selected individuals used as the comparison cohort; those with tuberculous meningitis and CNS involvement were excluded. The hazard ratio of ischemic stroke was 1.52 (95%CI = 1.21-1.91) in the TB group compared to the non-TB group; it was concluded that patients with TB were at increased risk for ischemic stroke for the subsequent three years following diagnosis [66].

Wu et al. researched the same subject; 5804 patients with non-CNS TB were grouped, and 5804 matched subjects without TB were put in another group. In the three-year follow-up, 3% of the TB group and 3.6% of the non-TB group had developed ischemic stroke. The hazard ratio of ischemic stroke was 0.92 (95%CI = 0.73-1.14; P =0.4299) in the TB group compared to the non-TB group. It was concluded that there was no increase in ischemic stroke in patients with non-CNS TB [67].

There is a paucity of data on this, and more research should be carried out. No peculiar differences in management guidelines have been published. As such, medical practitioners should ensure adequate treatment of the infectious process and prompt attention to the ongoing ischemic cerebrovascular accident.

Tuberculosis and other complications

Other rare cardiovascular complications have been mentioned in the literature, such as Rasmussen aneurysms, affectation of bronchial and non-bronchial systemic arteries, mediastinal fibrosis, and annular sub-valvular ventricular aneurysms [68].

Strengths and limitations

A strength of this review is that many original studies were reviewed, with results and conclusions adequately compared and contrasted. There was limited data on certain complications due to the rarity of such disorders. The relationship between TB and CVDs was reviewed in isolation, exempting other possible variables as that was the focus of this review.

## Conclusions

Cardiovascular complications of tuberculosis have a high morbidity and mortality rate. This article has elaborated on the specific complications, analyzed various original studies, and summarized the available epidemiological data. The medical and surgical management of the complications has also been discussed. This review has shed light on the intersection of two highly significant causes of mortality, TB and cardiovascular diseases. It will aid physicians in being better equipped to manage patients and reduce mortality, as it elaborates on both CVS complications with higher prevalence and those with lower incidence rates that can be more easily missed. There should be a high index of suspicion and inter-disciplinary involvement of the various required specialties in management. Patients who are successfully treated for active TB should continue to be monitored for long-term cardiovascular complications. Also, more studies should be conducted on the various cardiovascular complications in TB to improve management guidelines for affected patients and further reduce mortality.
